# Impact of genome assembly status on ChIP-Seq and ChIP-PET data mapping

**DOI:** 10.1186/1756-0500-2-257

**Published:** 2009-12-16

**Authors:** Nicolas Buisine, Laurent Sachs

**Affiliations:** 1 Department Evolution des Régulation Endocriniennes, Museum National d'Histoire Naturelle, 7, Rue Cuvier, 75231 Paris Cedex 05, France

## Abstract

**Background:**

ChIP-Seq and ChIP-PET can potentially be used with any genome for genome wide profiling of protein-DNA interaction sites. Unfortunately, it is probable that most genome assemblies will never reach the quality of the human genome assembly. Therefore, it remains to be determined whether ChIP-Seq and ChIP-PET are practicable with genome sequences other than a few (*e.g*. human and mouse).

**Findings:**

Here, we used *in silico *simulations to assess the impact of completeness or fragmentation of genome assemblies on ChIP-Seq and ChIP-PET data mapping.

**Conclusions:**

Most currently published genome assemblies are suitable for mapping the short sequence tags produced by ChIP-Seq or ChIP-PET.

## Background

In the past few years, next-generation sequencing technologies have fuelled a plethora of studies providing genome wide profiling of transcription factor DNA binding sites (TFBS) [1,2] and histone modifications [3-6]. These data are of highly fundamental and applied relevance, as exemplified by the recent ChIP-Seq based profiling of 15 key stem cell-specific transcription factors binding sites, in mouse [2]. These technologies, which combine reduced cost, speed and effectiveness, have been primarily developed to be used together with high quality genome assemblies (although not necessarily complete), such as those of human [1], mouse [2], drosophila [7], yeast [8] and arabidopsis [9].

ChIP-Seq is a new application of chromatin immuno-precipitation technologies and is particularly adapted to map protein-DNA contacts across the genome. To this end, chromatin is fragmented and immuno-precipitated with an antibody raised against a DNA binding protein and one end of each purified DNA fragment is sequenced with an ultra-high throughput sequencer. ChIP-PET [10,11] is similar to ChIP-Seq except that the two ends are sequenced, thus providing greater specificity in mapping the reads to the genome.

The number of sequence tags at any genomic location is a quantitative value, which reflects the local enrichment of the DNA-bound protein, and clusters of tags (peaks) are used to define TFBS.

Sequence tags mapping, by which the short sequence reads (tags) produced by ultra-high throughput sequencing are mapped onto a reference genome sequence, is a critical step since it will dictate the outcome of downstream analyses. Thus, the bioinformatic analysis of ChIP-Seq and ChIP-PET data implicitly relies on the quality of the reference genome assembly both for sequence tags mapping and for mining the relative position of DNA binding sites with other functional and structural components of the genome [1,5]. Unfortunately, most genome assemblies correspond to draft genome sequences composed of many scaffolds and containing numerous assembly gaps (unsequenced regions), which can potentially impair sequence tags mapping. In this paper, we model ChIP-Seq and ChIP-PET data sequence tags mapping on draft or incomplete genome sequences. Beyond the obvious fact that if the binding sites occur in the known parts of the genome they will be detected, our data suggest that the state of a genome assembly has a limited impact on sequences tags mapping and that most genome assemblies are readily usable for ChIP-Seq and ChIP-PET analysis.

## Findings

### 1. State of assembly

Most genome assemblies released to date have not benefited from extensive curation efforts and do not reach the high quality standard of a few model organisms, for which the genome sequences are almost complete and the sequence of individual chromosomes has been reconstructed. Indeed, the human and mouse genome assemblies are respectively composed of 24 chromosomes and 22 chromosomes together with a few unmapped scaffolds. In other cases, it is instead a draft sequence, almost always fragmented into many scaffolds (see Additional file 1: Figure S1, and Additional file 2: Table S1). For example, although the platypus genome assembly is about the same size as the mouse genome assembly, it is composed of ~291,000 scaffolds with an average size of 6.8 kb compared to 22 chromosome of ~60 to 200 Mb. Surprisingly, a few unpublished genome assemblies are less fragmented than published ones. This is the case, for example, of the xenopus genome assembly, which is intermediate between mouse's and platypus', with a total of ~20,000 scaffolds and an average scaffold size of 77 kb. Importantly, a limited subset of 1,440 scaffolds represents ~90% of the assembly, which further shows the relatively low level of fragmentation of this assembly. This contrasts sharply with platypus where ~90% of the assembly is represented by 35,779 scaffolds (genome assemblies available at ENSEMBL web site [12]).

### 2. Completeness of assemblies

The sequenced fraction of published assemblies is also quite variable and ranks from ~70% to ~100% (for *Ciona intestinalis *and *Cenorhabditis elegans*, respectively). Importantly, almost all (98.5%) Ns found in genome assemblies released to date are part of stretches of at least five consecutive Ns. This means that virtually all Ns actually correspond to assembly gaps (unsequenced regions) rather than isolated sequencing ambiguities. In published draft sequences, unsequenced gaps represent 2.94% to 28.3% of the assembly (for *Monodelphis domestica *and *Takifugu rubripes*, respectively). Thus, they are a potential pitfall for ChIP-Seq and ChIP-PET sequence reads mapping as many DNA binding sites may be missed. Therefore, these data suggest that although ChIP-Seq and ChIP-PET proved to have a remarkable resolution in mouse and human, their application to other sequenced genomes remains an open question. A key point is that currently available (as well as future) assemblies will certainly not benefit from curation efforts similar to those of a few model organism and are unlikely to reach their quality standard. This is a severe limitation to the application of ChIP-Seq and ChIP-PET to other genomes. It is therefore critical to assess whether one can make use of ChIP-Seq technologies with the existing genome assemblies. We addressed this issue by modelling ChIP-Seq and ChIP-PET data mapping *in silico*.

### 3. Mapping simulations

#### 3.1 Rationale

**Figure 1:**
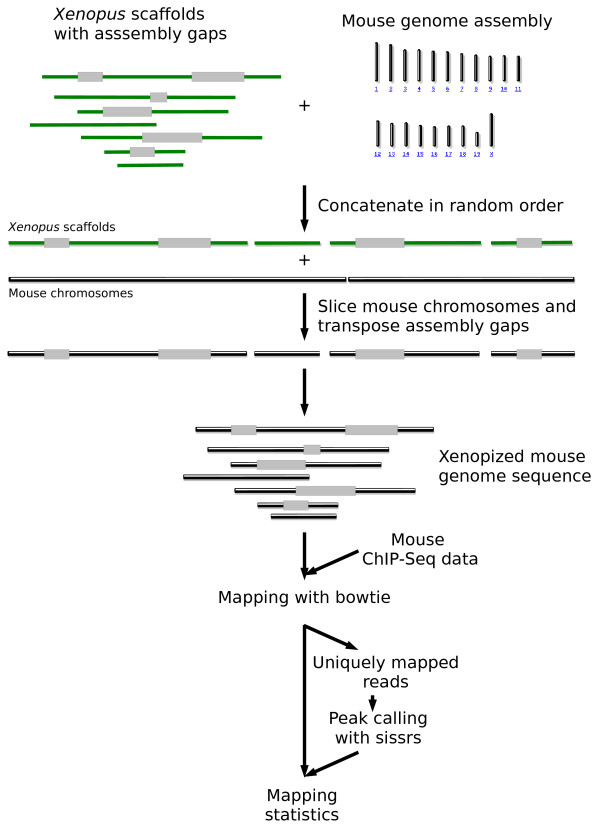
**Principle of *in silico *simulations of ChIP-Seq tags mapping with gapped genome assemblies**. In this example, the *Xenopus *scaffolds and the mouse chromosomes were concatenated in a random order and placed along each other. Each *Xenopus *assembly gap was then transposed onto the mouse genome, which was further sliced in order to reflect the fragmented nature of the *Xenopus *genome assembly. ChIP-Seq sequence reads were mapped with bowtie on the 'xenopized' mouse genome. Sequence reads mapping at multiple locations were discarded and peak calling was carried out with sissrs. The process is reiterated 20 times for statistical robustness. With ChIP-PET, the process is similar except that the two ends of each sequence read are mapped.

*In silico *simulation of ChIP-Seq data mapping assumes to model the distribution of sequence reads frequency and depth. These models can be complex to build in part because the DNA-binding domain of a transcription factor is susceptible to bind to different sequence. Furthermore, the distribution of assembly gaps may be specific to each assembly since it depends on the software algorithm, sequencing libraries used to build it and the underlying structure of the genome. Therefore, in order to rule out inaccurate models, we undertook a brute force approach and turned top quality genome assemblies into ones that reflect those at different levels of assembly ('xenopization', 'batization', 'bovization'...), by fragmenting and introducing assembly gaps taken from a query assembly into the human or mouse genome assemblies (see below). Thus, xenopization fragments human/mouse genome assembly in a manner similar to that of *Xenopus tropicalis*, bovization to that of *Bos taurus *and so on (Figure 1). We then scored the mapping efficiency of the mouse ChIP-Seq and human ChIP-PET data with the modified assemblies (see Methods). One can then assess the potential impact of the completeness of genome assembly onto human and mouse ChIP-Seq and ChIP-PET data mapping. Simply put, the question being asked is what would be the ChIP-Seq and ChIP-PET mapping outcome if the reference sequences (from human or mouse assembly) were similar to those of bat or xenopus? By extension, this can be used to estimate the probable success of ChIP-Seq and ChIP-PET mapping with the corresponding genome assembly.

#### 3.2. Mapping of ChIP-Seq data

To this end, we first used the ChIP-Seq datasets obtained for 15 mouse embryonic stem cell specific transcription factors (plus an additional GFP control, [2]). Using the mapping simulation pipeline (see Methods), we surveyed the genome assemblies available at the ENSEMBL website (statistics available in Additional file 2: Table S1) and measured the ChIP-Seq data mapping success of all 15 transcription factors datasets (Additional file 3: Table S2).

The rate of successful mapping of ChIP-Seq tags ranks from 100% for *Arabidposis thaliana*, which has almost no assembly gaps, to 46.82% for *Felis catus*, the genome of which is currently partially sequenced (Additional file 3: Table S2). Not surprisingly, DNA binding sites are missed less often (by 3% to 5%) than isolated tags. Among published genome sequences, the mapping success observed with *Tetraodon nigroviridis *assembly parameters proved surprisingly low with ~25% missed tags. This probably reflects the fact that this assembly is composed of ~30% assembly gaps. Overall, the unsequenced fraction of an assembly (*i.e*. the cumulated gap size) is a good estimator of the ChIP-Seq data mapping outcome, although it tends to overestimates it by ~5-10%. We also note that the rate of successful mapping is very similar between the 15 transcription factors tested, which have clear distinct DNA binding properties (number of sites across the genome, tag density per DNA binding site, [2]).

#### 3.3. Mapping of ChIP-PET data

We next assessed mapping success with the ChIP-PET data sets obtained with human p53 and STAT1 transcription factors [10]. The difference between this dataset and the ChIP-Seq datasets is two fold: 1) the ChIP-PET sequencing depth is somewhat lower and 2) the two ends of each chromatin fragment are being sequenced (di-tags). To this end, we adapted to procedure detailed above to the ChIP-PET data. Results were virtually identical to those obtained with ChIP-Seq (Additional file 4: Table S3). The successful mapping of the two ends ranks from 99.5% for *Arabidposis thaliana *to 42.06% for *Felis catus*. Failure to map the two ends follows an opposite trend, from 0 for *Danio rerio *to 48.77% for *Dasypus novemcinctus*. Single end mapping ranks from 0.02% to 12.5%. The fragmentation of a genome assembly has a limited impact on ChIP-PET data mapping efficiency. For example, the genome assembly of *Takifugu rubripes *is composed of ~7,000 scaffolds and that of *Xenopus tropicalis *of ~20,000 scaffolds, but they display similar one and two ends mapping success (Additional file 4: Table S3).

## Conclusions

Collectively, these results show that assembly gaps and fragmentation of the mouse and human genome sequence do not prevent mapping of ChIP-Seq and PET-ChIP data. By extension, one can infer that most genome assemblies are suitable for ChIP-Seq and ChIP-PET analysis. Also, the fact that a draft genome sequence is published does not guarantee a high mapping efficiency, as exemplified by *Tetraodon nigroviridis*, for which mapping efficiency was found surprisingly low.

Our conclusions can be extended to research areas based on high throughput sequencing other than mapping of protein-DNA interaction sites, such as genome resequencing, SNP detection and other di-tag sequencing technologies.

## Methods

### Statistics of genome assemblies

Statistics of genome assemblies were computed with a simple python script.

### Mapping assessment pipeline

For a given ChIP-Seq dataset, the mapping simulation pipeline described below was run for the genome assemblies available at ENSEMBL website.

Mouse chromosomes were randomly joined together in order to form an artificially long chromosome (ALC). Each scaffold of the test assembly was then randomly selected and its gap content transposed into the left end of the ALC, which was further truncated in a fragment of the scaffold' size (Figure 1). This process was iterated over all the scaffolds of the test assembly. The resulting assembly (*e.g. *xenopized assembly, if the test assembly is that of *Xenopus tropicalis*) was used as a reference to map ChIP-Seq datasets [2] (GEO accession number GSE11431, 26 bp sequence reads) with bowtie [13] (version 0.10.0), using stringent parameters (-q -l24 -m 3, *i.e. *up to two mismatches; quality values are ignored). Sequence reads mapping at multiple genomic locations were discarded. Transcription factor binding sites were detected ("Peak calling", Figure 1) with sissrs [14], run with a false discovery rate of 1‰. The whole process was reiterated 20 times for statistical robustness. This dataset correspond to 15 transcription factors (plus one control) which have different DNA binding properties and number of binding sites, and thus represent an ideal benchmark tool. All the datasets were initially mapped on the mouse genome in order to benchmark the mapping and peak calling parameters. We found a similar number of TFBS to those reported by [2]. For mapping assessment of ChIP-PET data (from [10]), the process is essentially the same, except that the two ends of each PET are mapped and that assembly gaps are introduced in the human genome.

Of notes, this procedure does not ask directly whether the query assembly is suitable for ChIP-Seq mapping, rather it scores the mapping efficiency of the ChIP-Seq data if they had been carried out on the subject assembly fragmented and containing as many assembly gaps as in the query. The procedure was encapsulated in a python script, using the "random" built-in module. Crucially, this module uses Mersenne Twister as the core generator, which is probably the most extensively tested and reliable random number generator.

## Competing interests

The authors declare that they have no competing interests.

## Authors' contributions

NB build the mapping simulation pipeline and ran all the analysis. NB analysed the data and NB and LS wrote the manuscript.

## Supplementary Material

Additional file 1 **Figure S1**. Genome sequences are often fragmented in many scaffolds containing unsequenced gaps. For each genome assembly available at ENSEMBL, the size and the unsequenced percent of each scaffold has been plotted. Click here for file

Additional file 2 **Table S1**. Statistics of genome assemblies. For a few species (grayed name), the estimated ChIP-Seq and ChIP-PET mapping efficiency is particularly low. Click here for file

Additional file 3 **Table S2**. Outcome of simulated ChIP-Seq mapping. Click here for file

Additional file 4 **Table S3**. Outcome of simulated ChIP-PET mapping. Click here for file
