# Supplementary material for: Impact of genome assembly status on ChIP-Seq and ChIP-PET data mapping
Source: BMC Res Notes. 2009 Dec 16;2:257. doi: 10.1186/1756-0500-2-257 (PMC2804576; doi:10.1186/1756-0500-2-257)
Supplement: Additional file 1 — Figure S1. Genome sequences are often fragmented in many scaffolds containing unsequenced gaps. For each genome assembly available at ENSEMBL, the size and the unsequenced percent of each scaffold has been plotted. [file 1756-0500-2-257-S1.PDF]

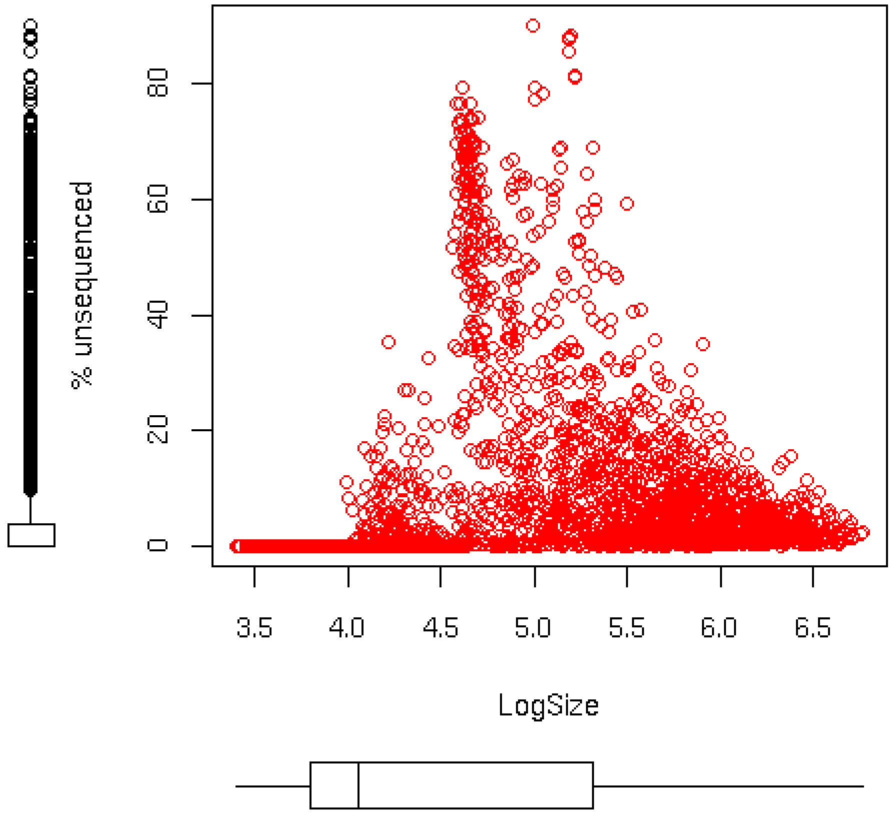

Aedes aegypti

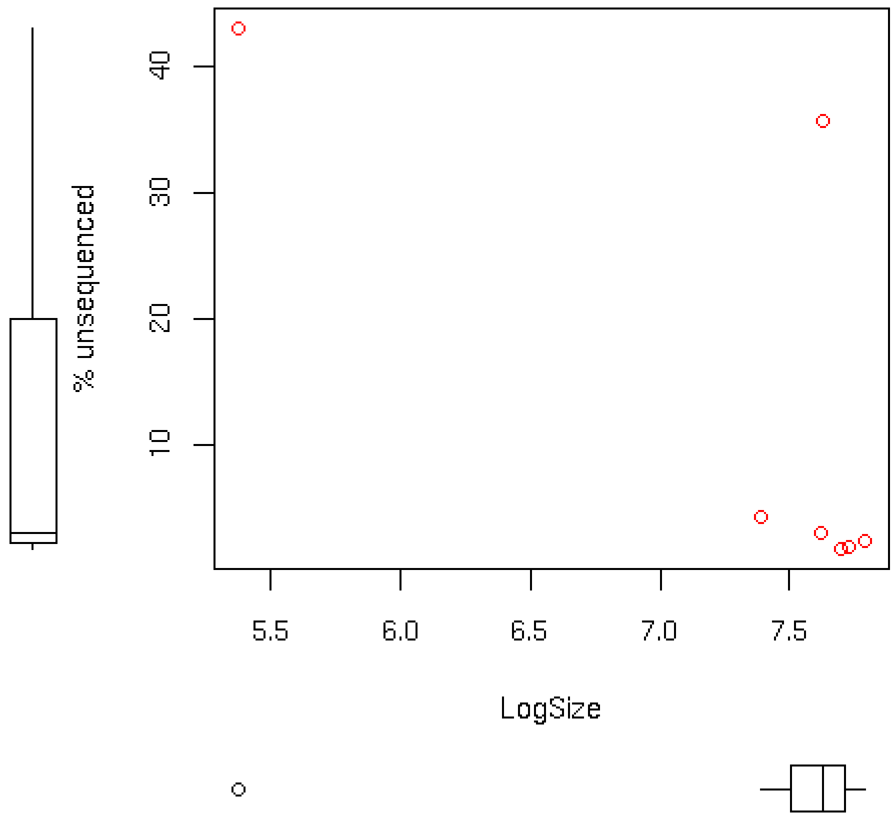

Anopheles gambiae

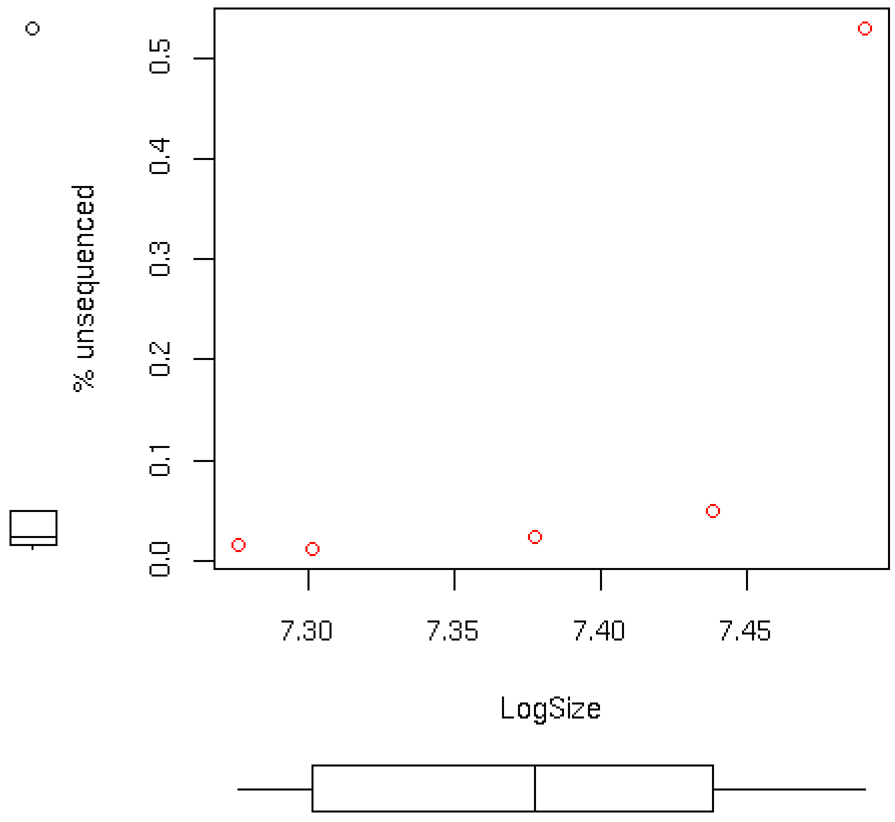

Arabidopsis thaliana

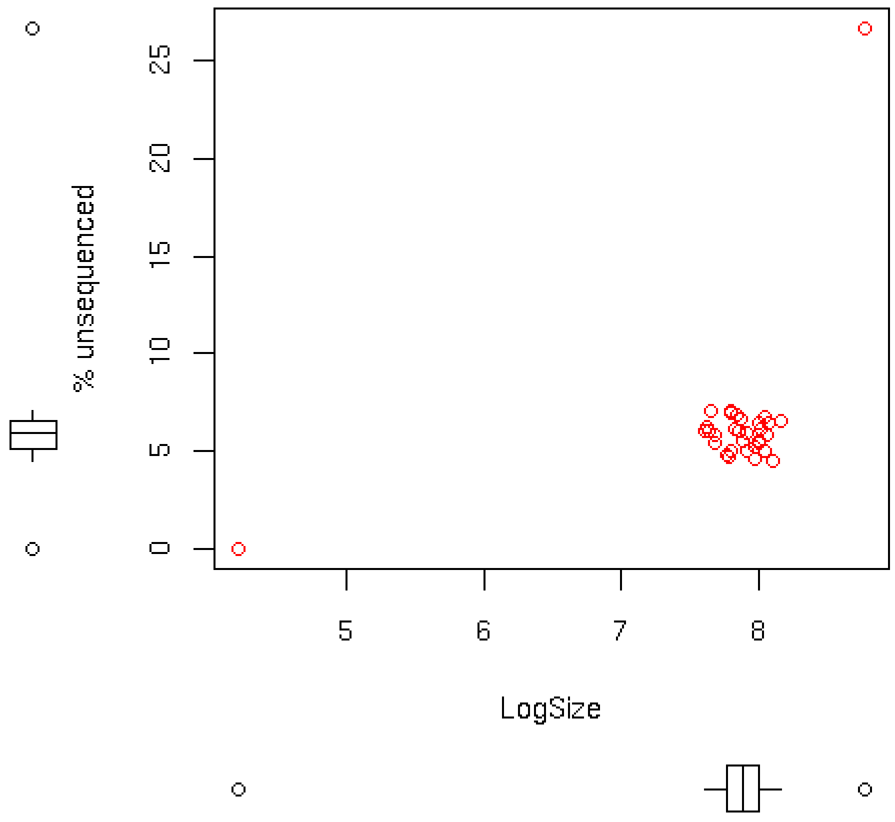

Bos taurus

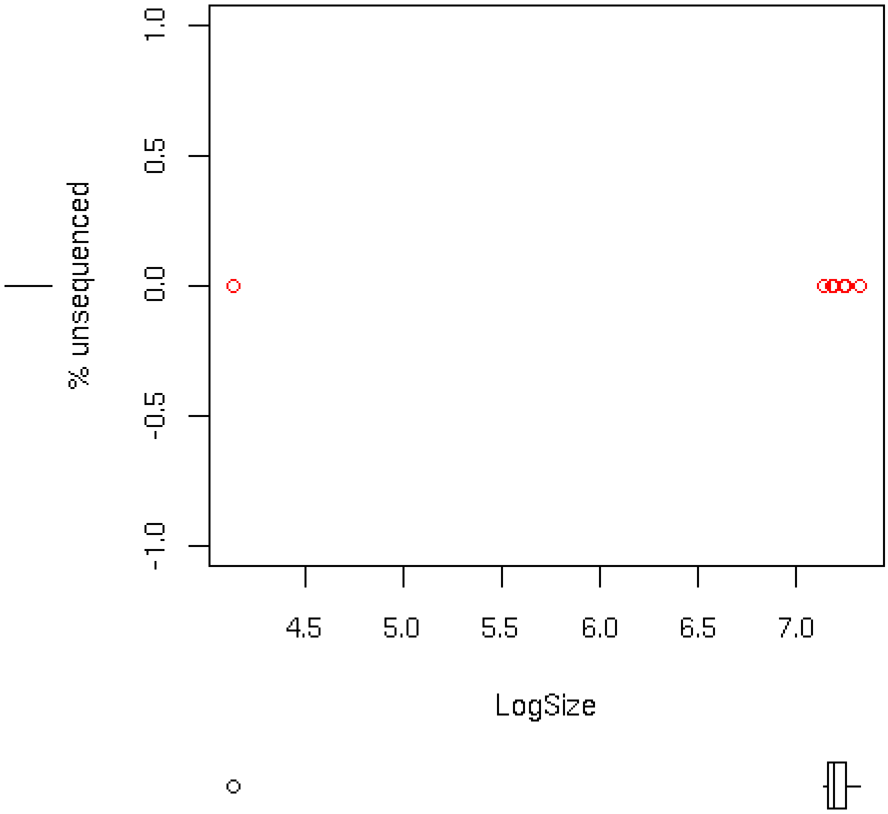

Caenorhabditis elegans

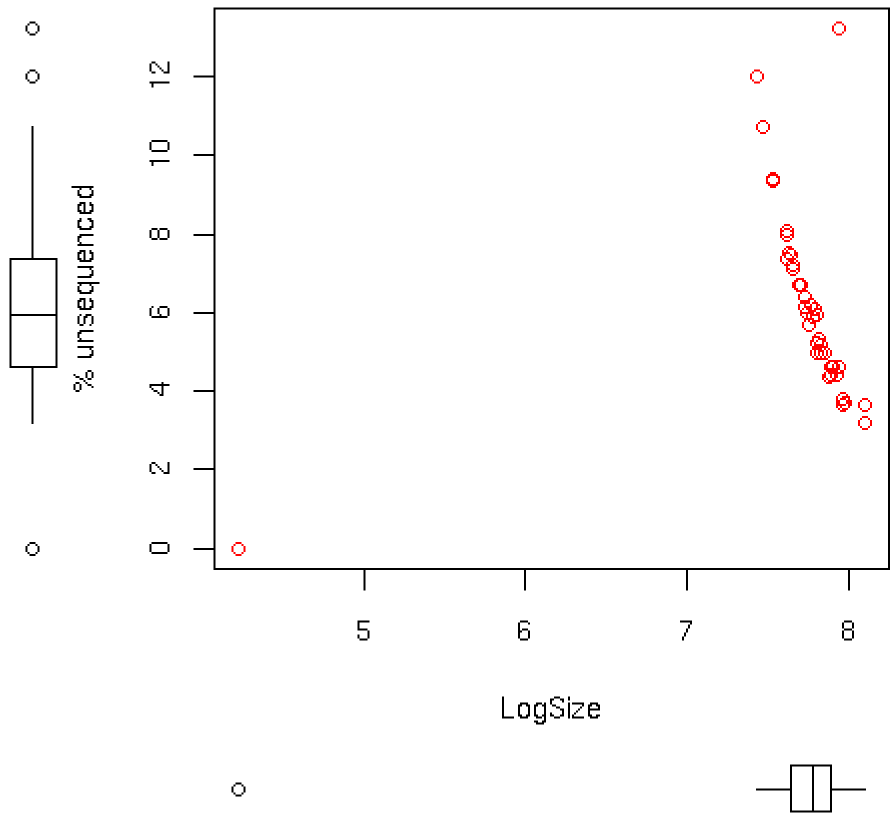

Canis familiaris

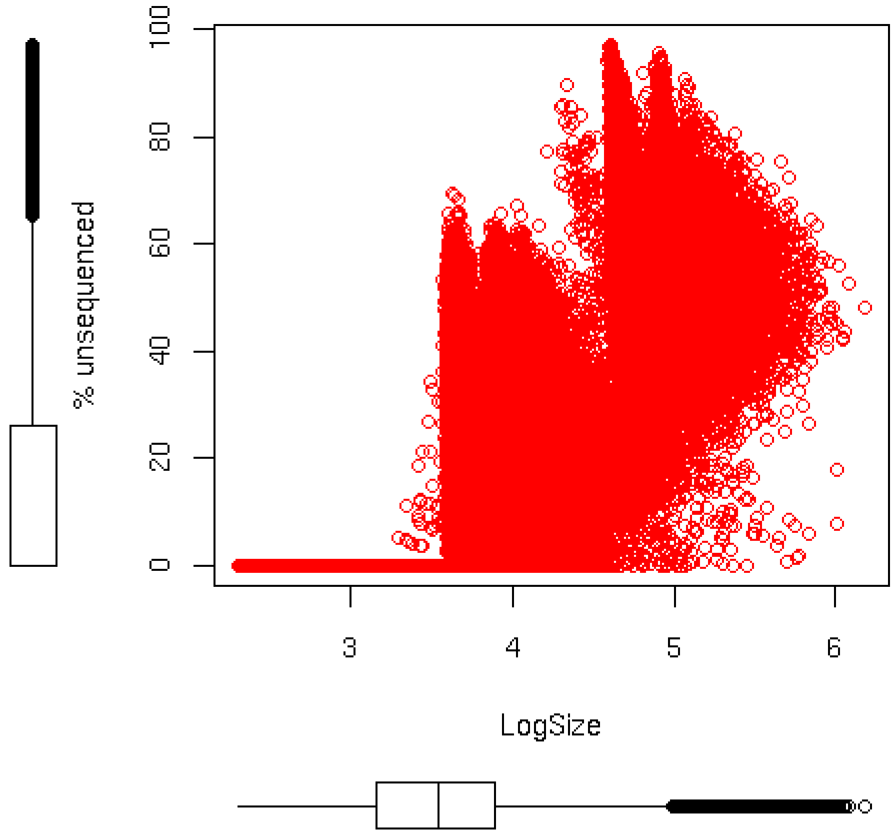

Cavia porcellus

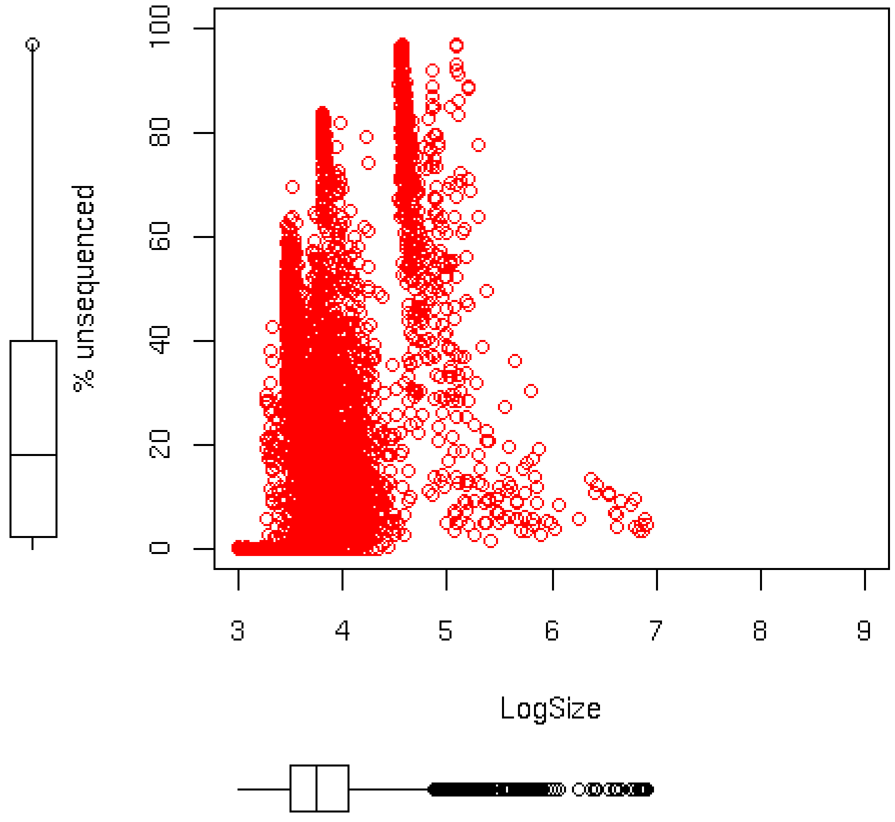

Ciona intestinalis

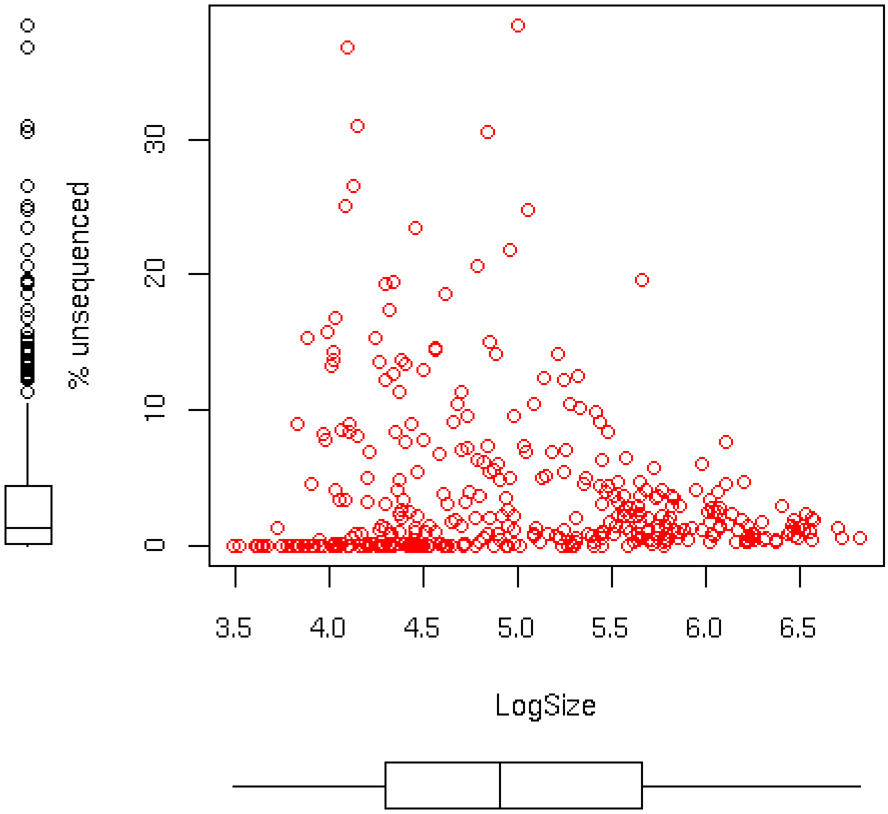

Ciona savignyi

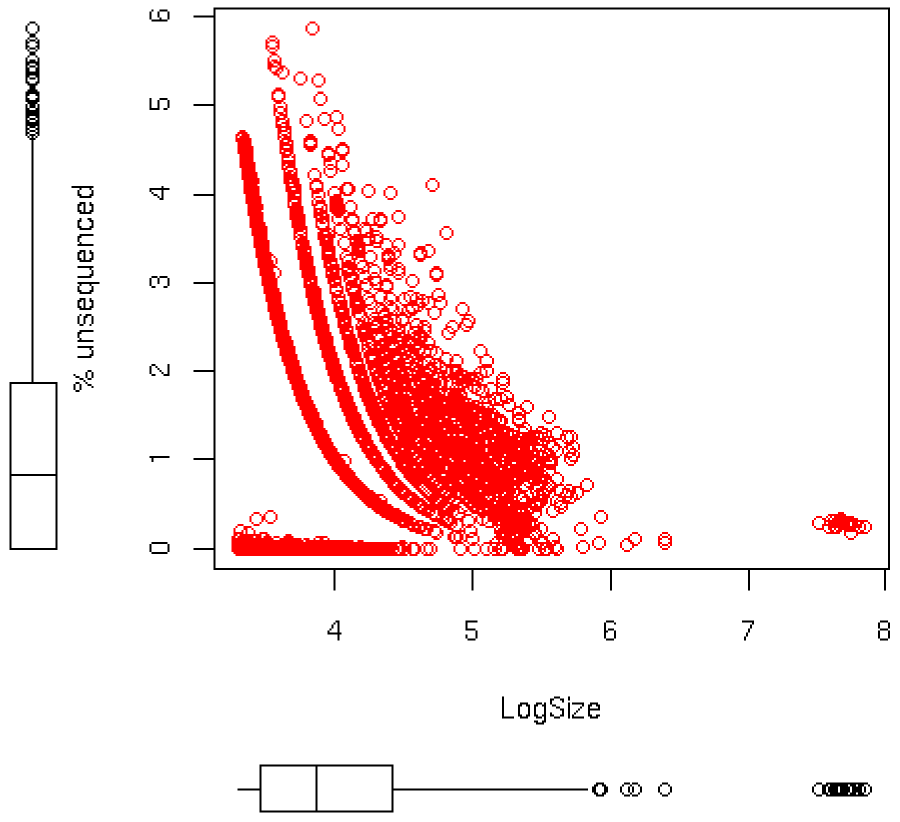

Danio rerio

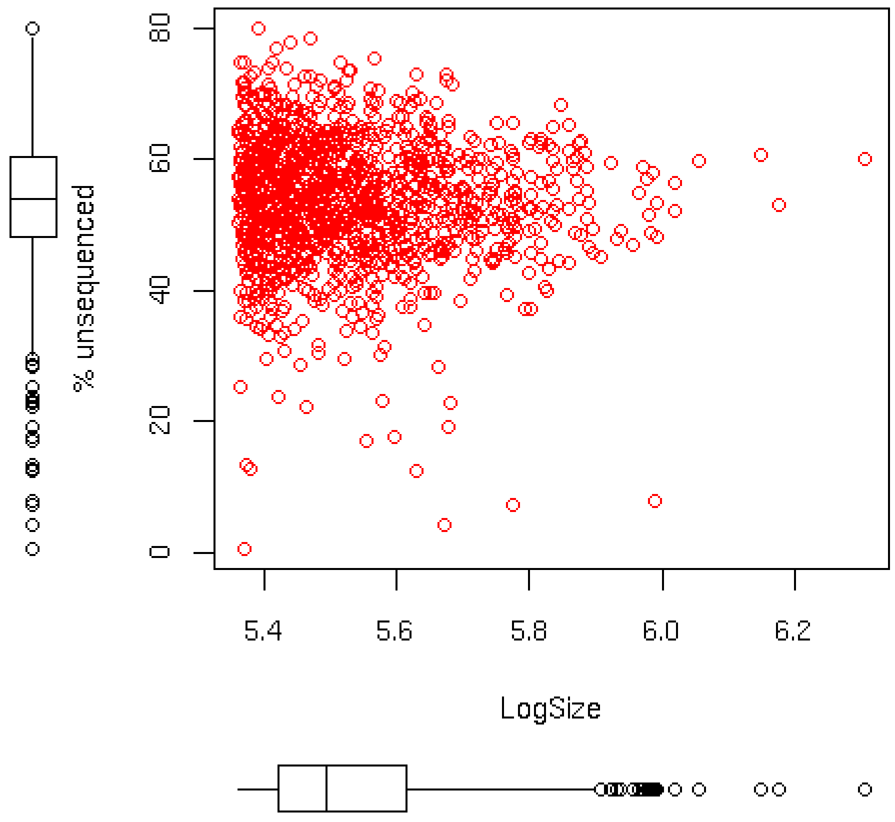

Dasypus novemcinctus

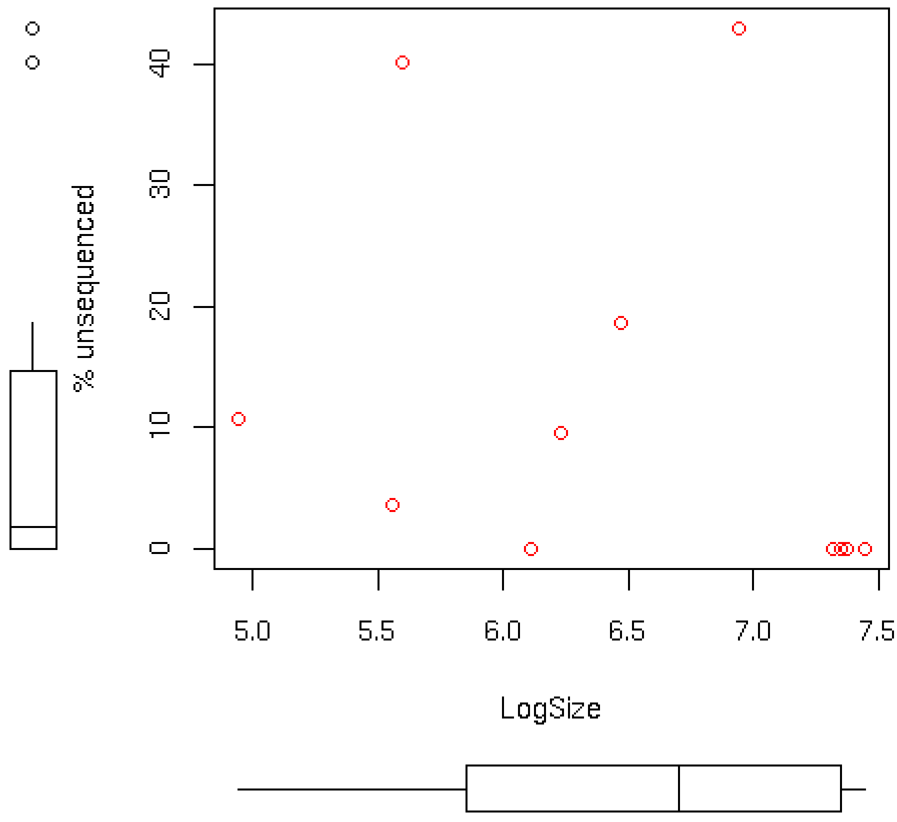

Drosophila melanogaster

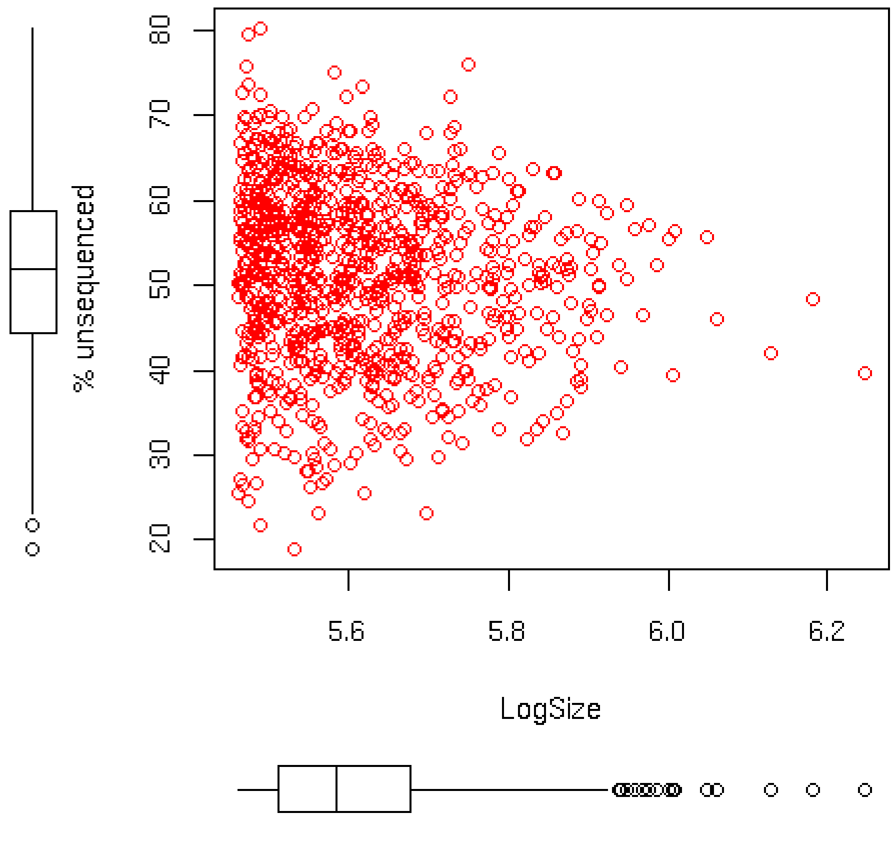

Echinops telfairi

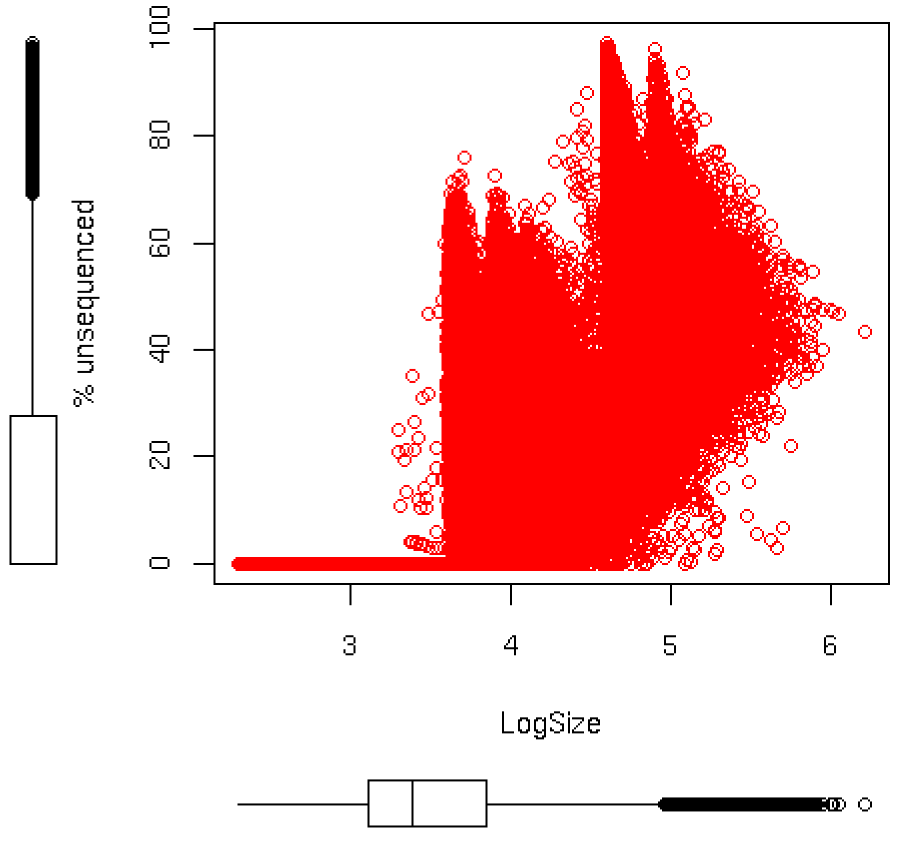

Erinaceus europaeus

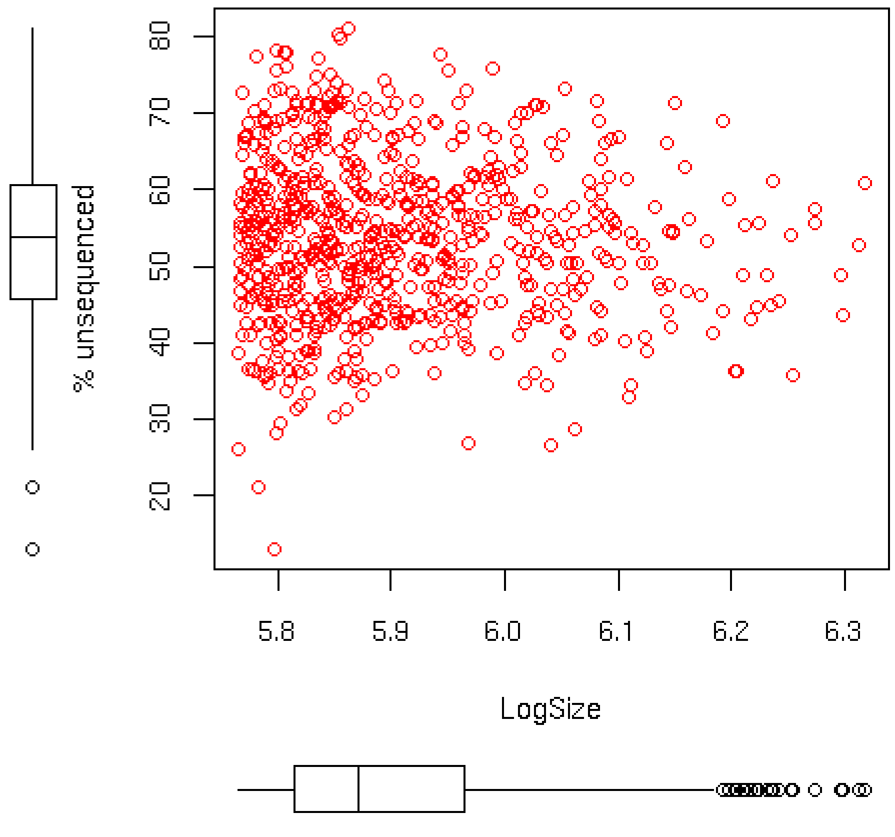

Felis catus

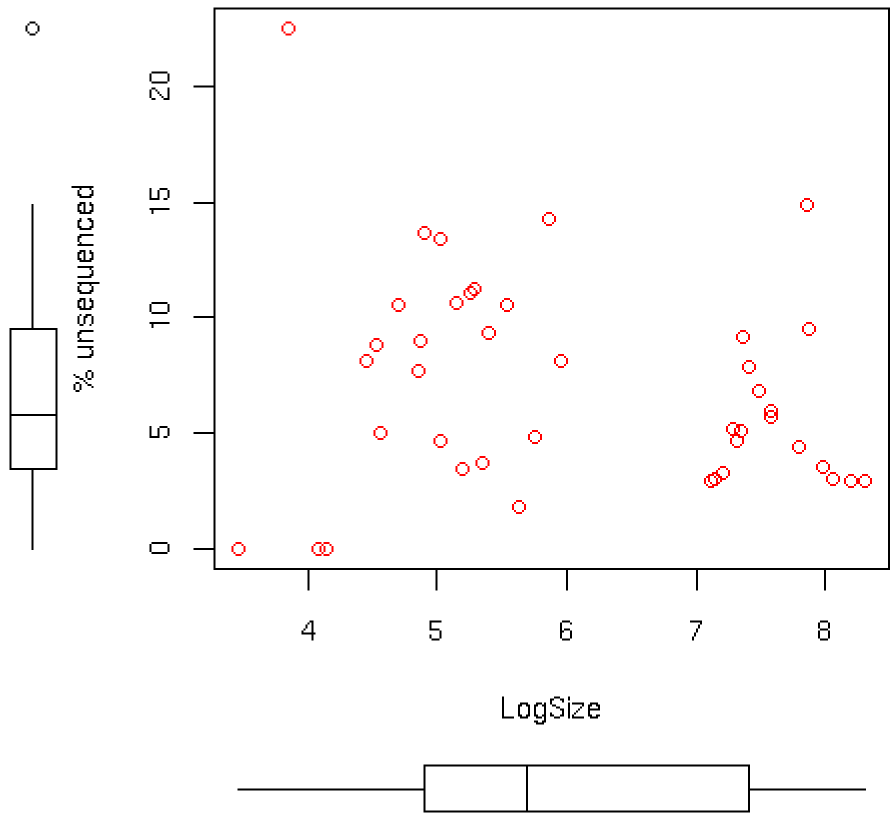

Gallus gallus

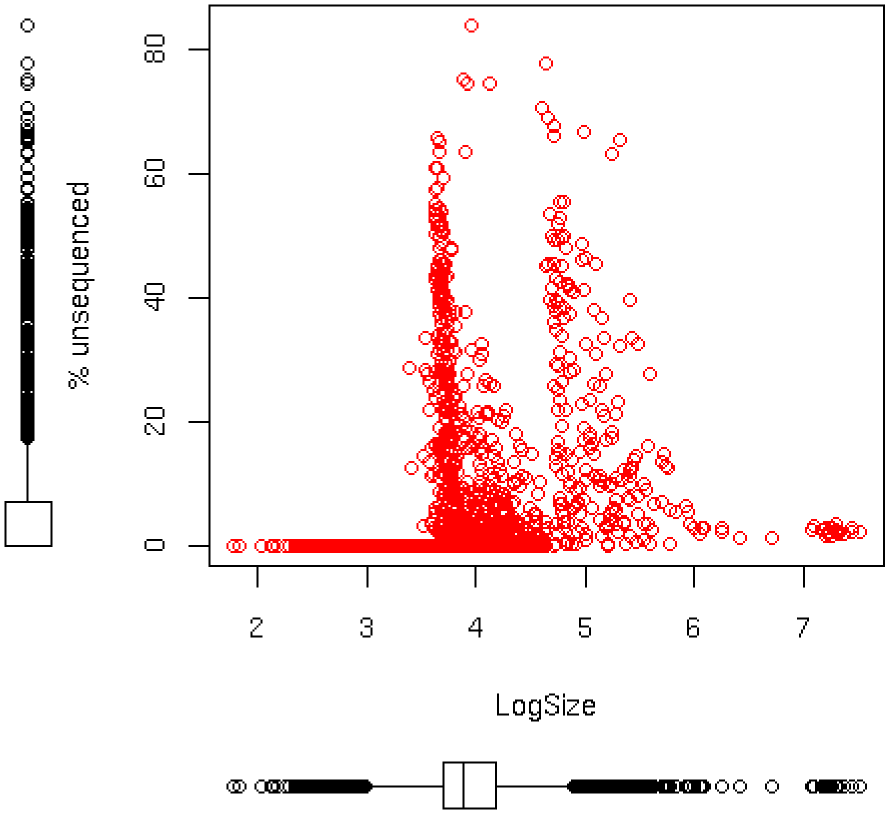

Gasterosteus aculeatus

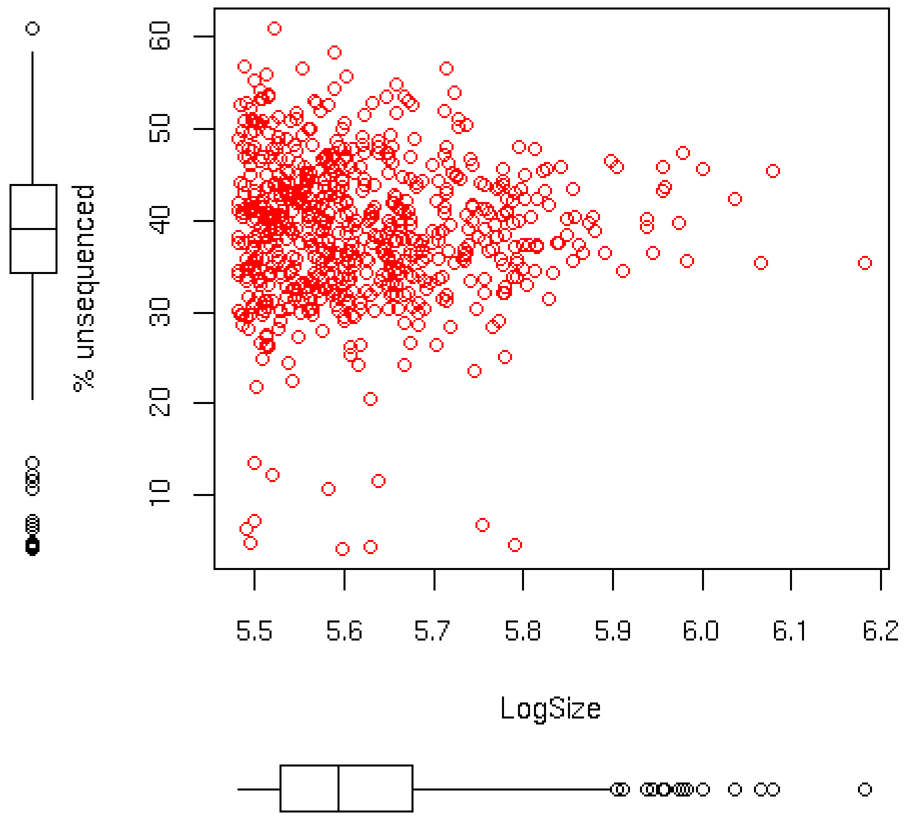

Loxodonta africana

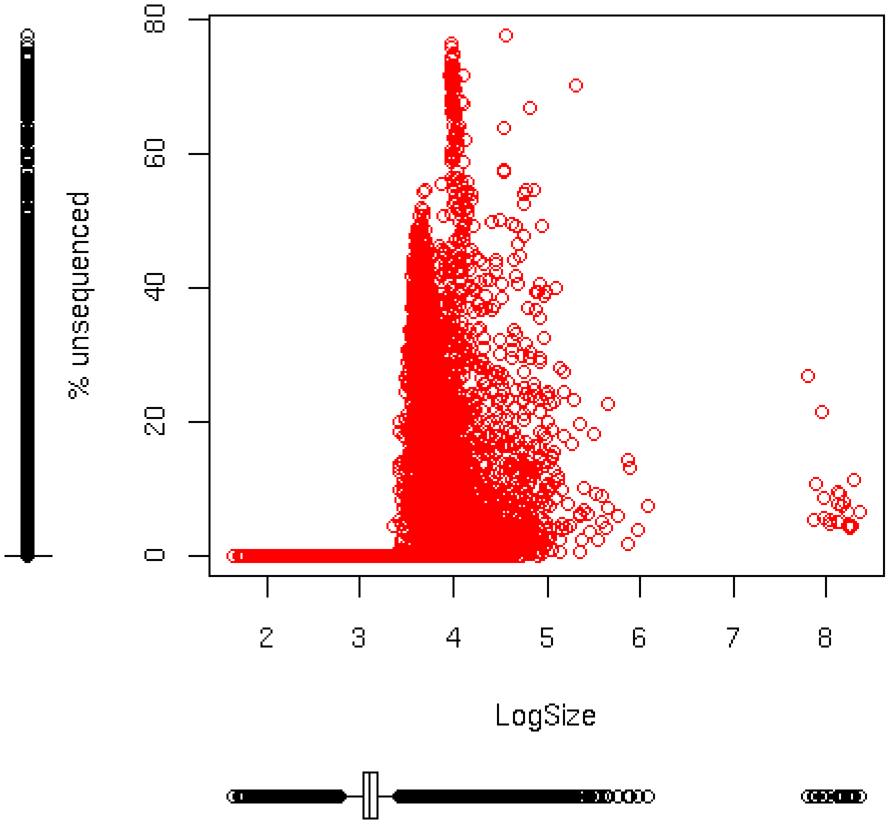

Macaca mulatta

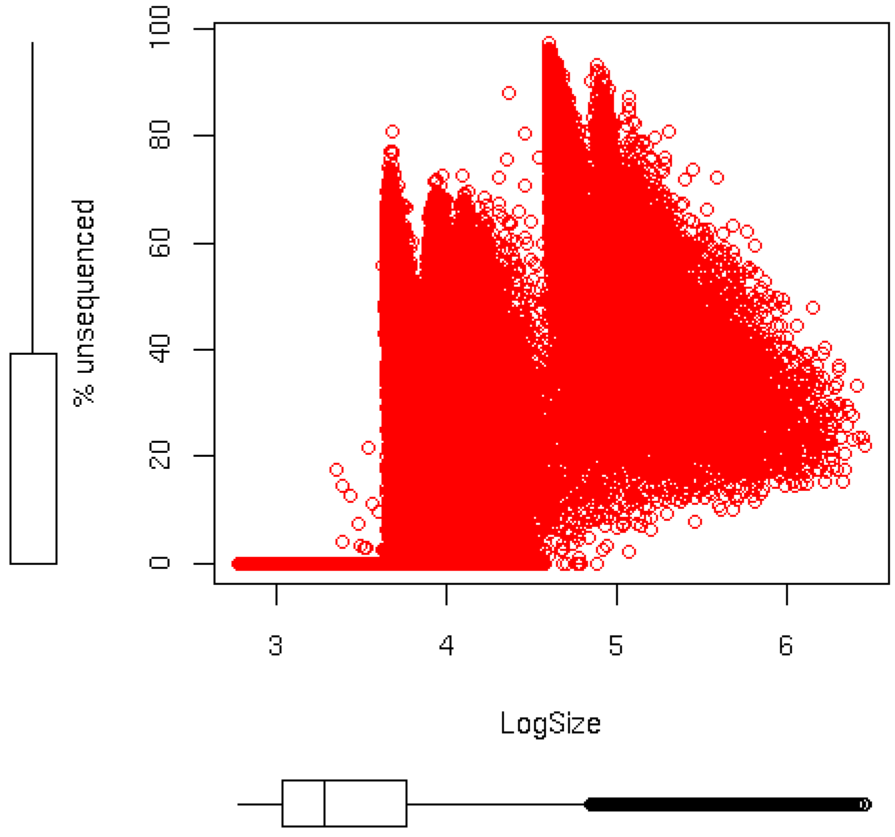

Microcebus murinus

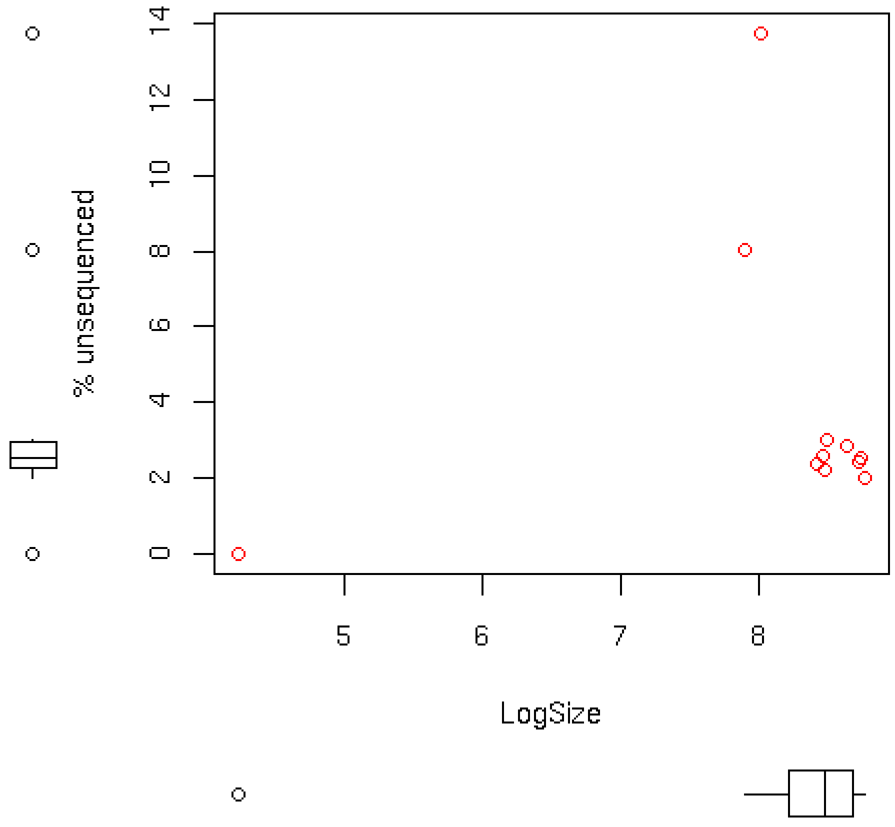

Monodelphis domestica

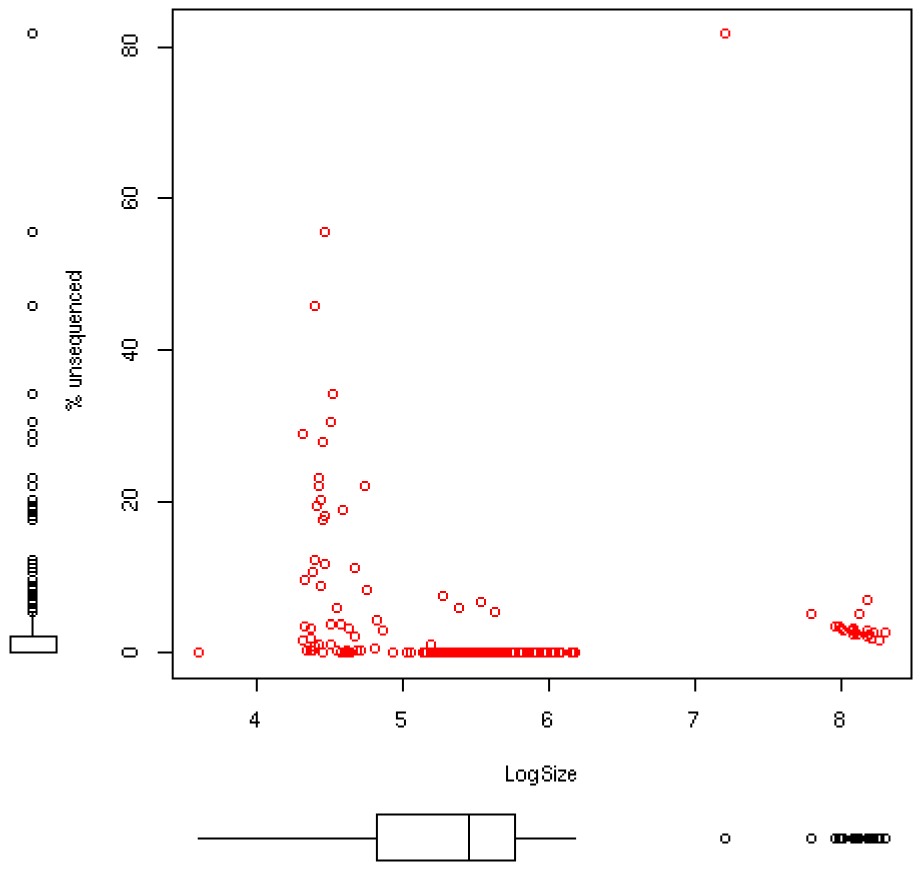

Mus musculus

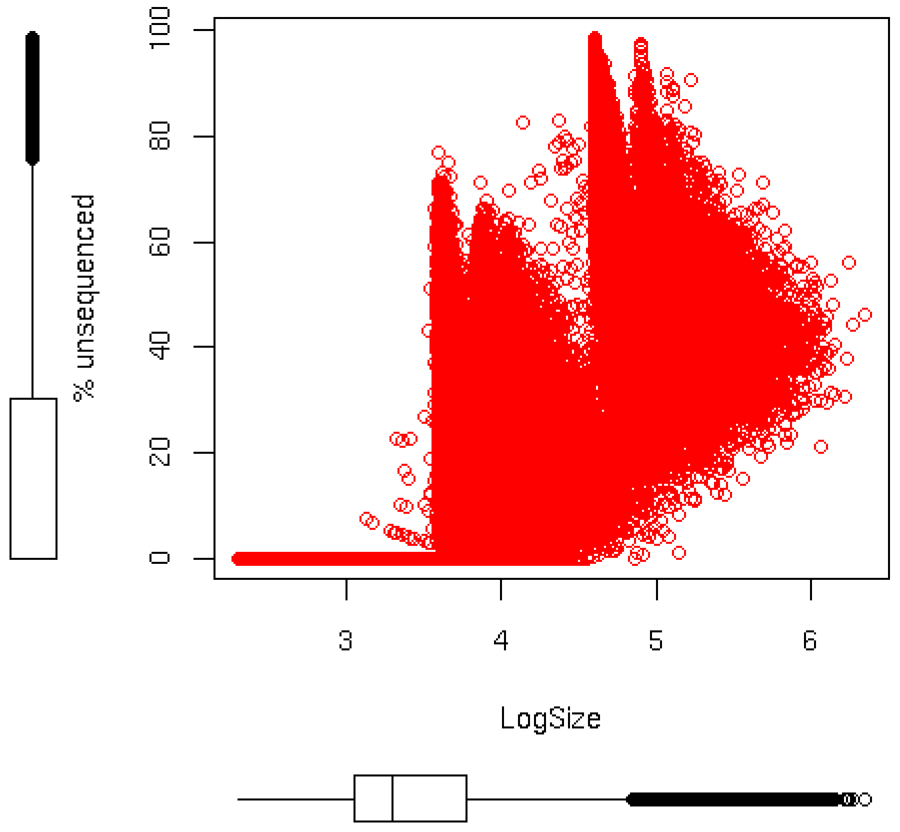

Myotis lucifugus

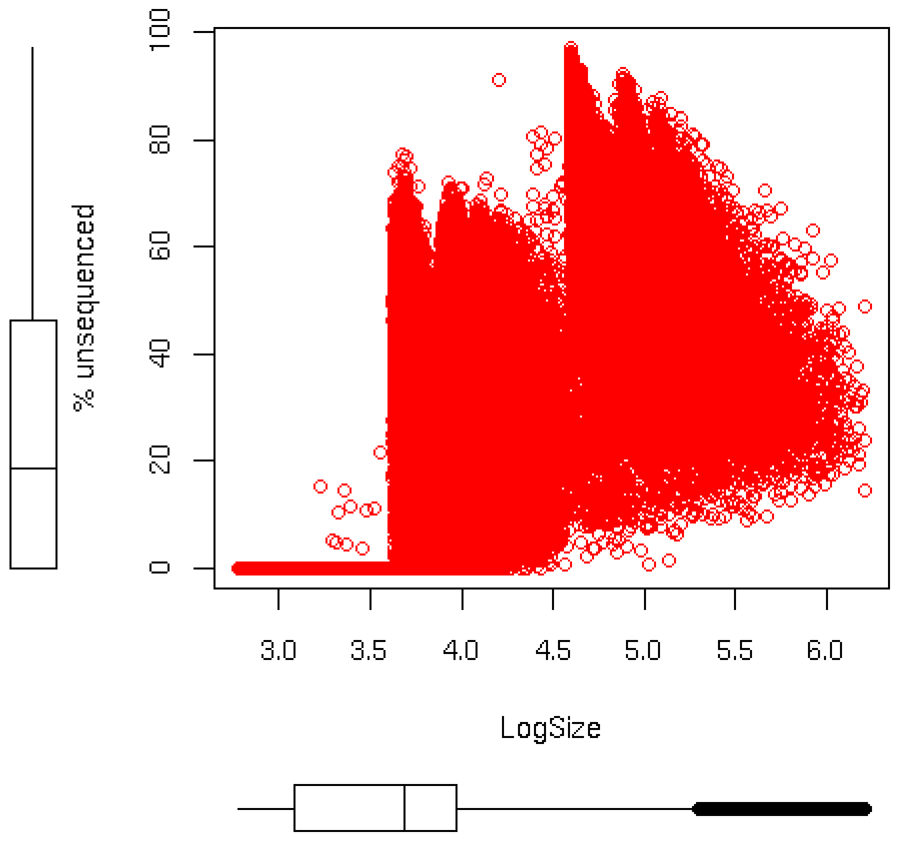

Ochotona princeps

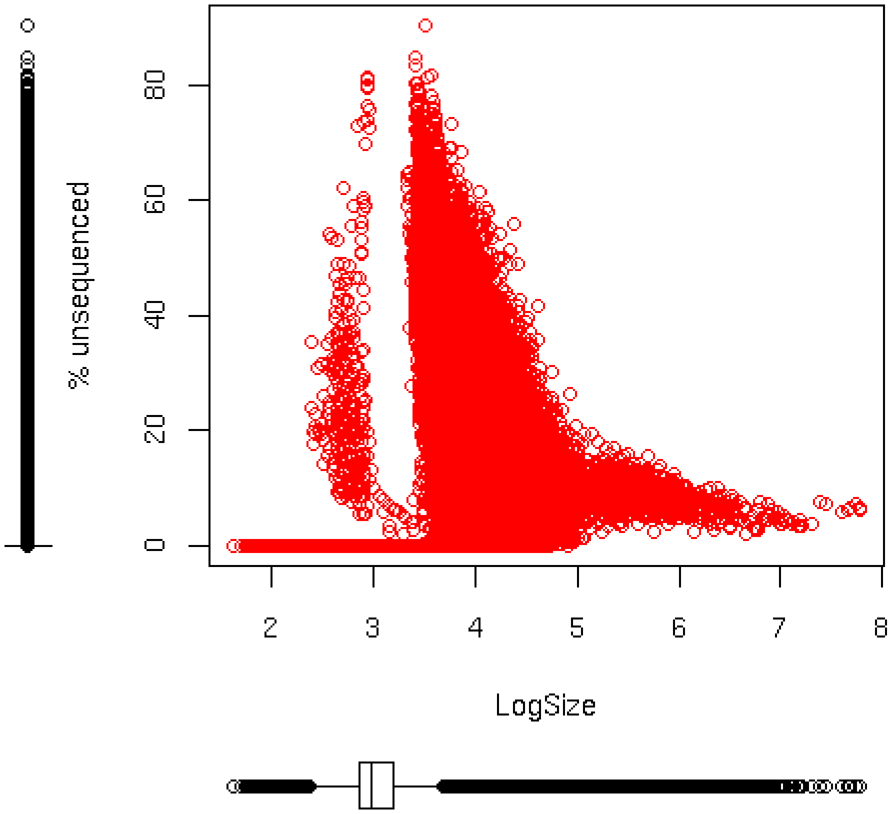

Ornithorhynchus anatinus

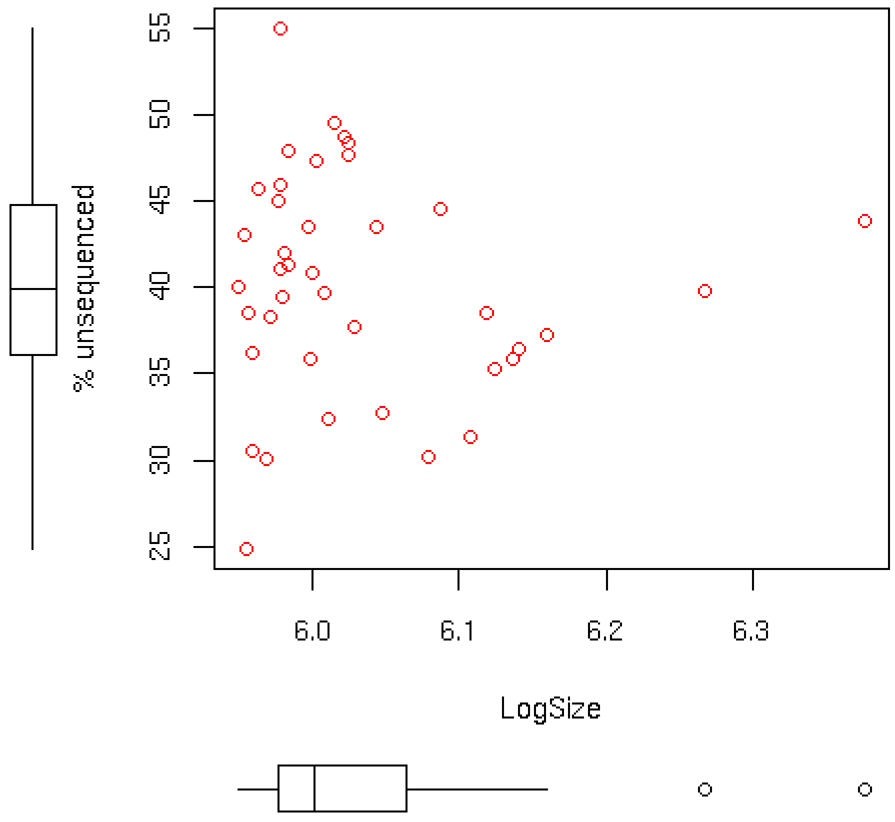

Oryctolagus cuniculus

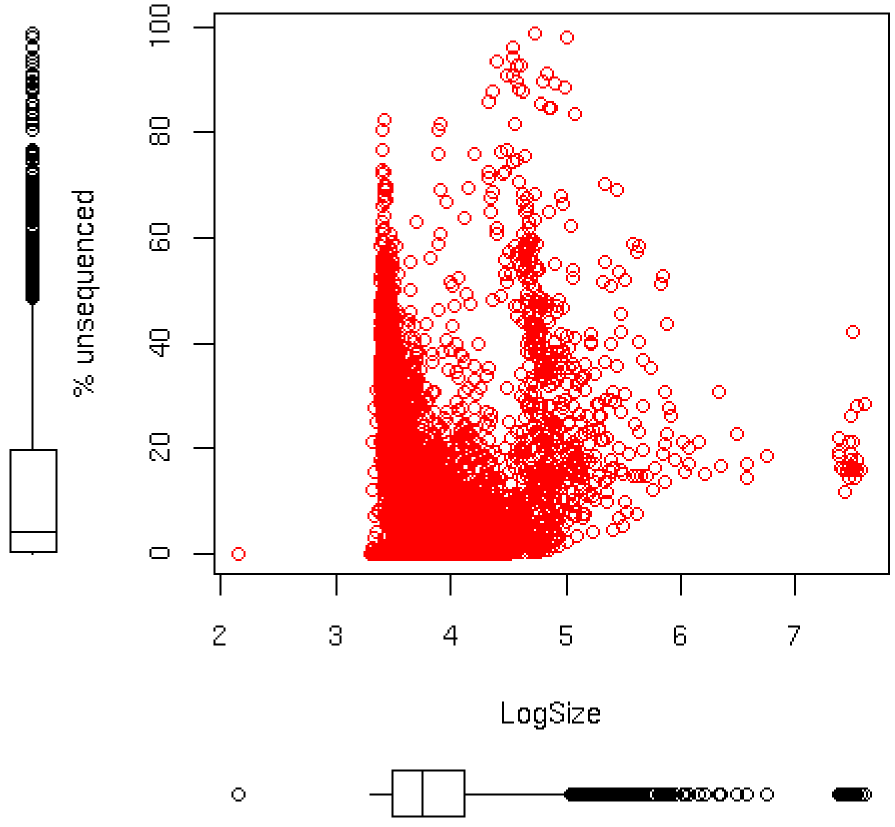

Oryzias latipes

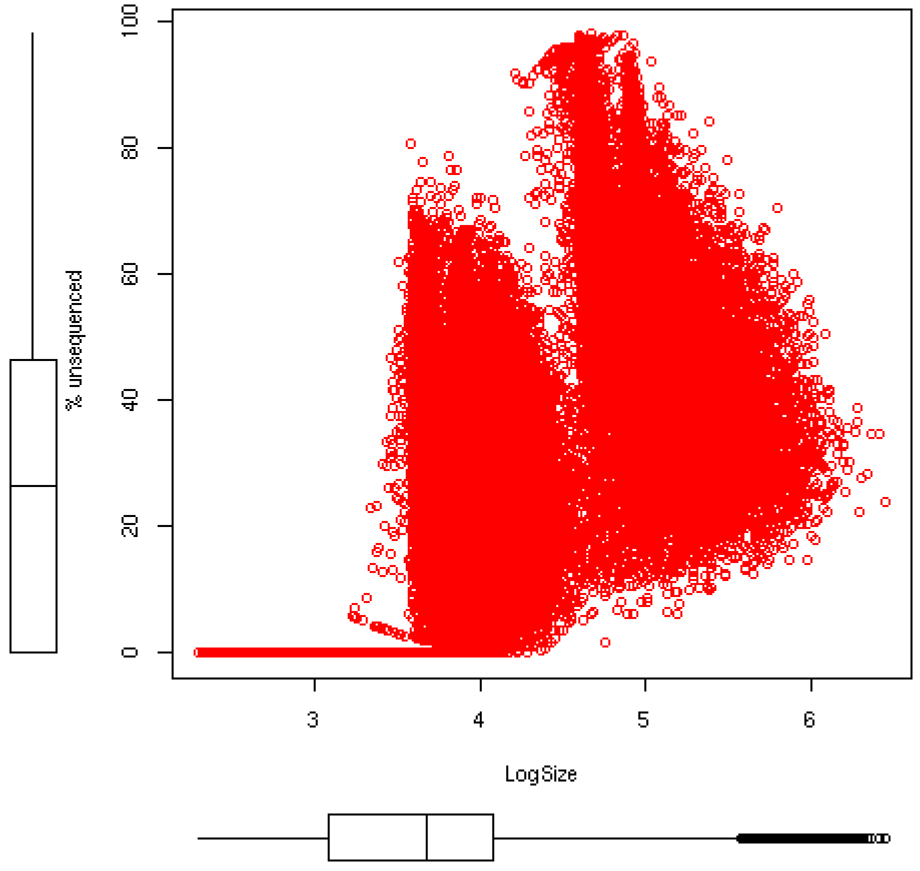

Otolemur garnettii

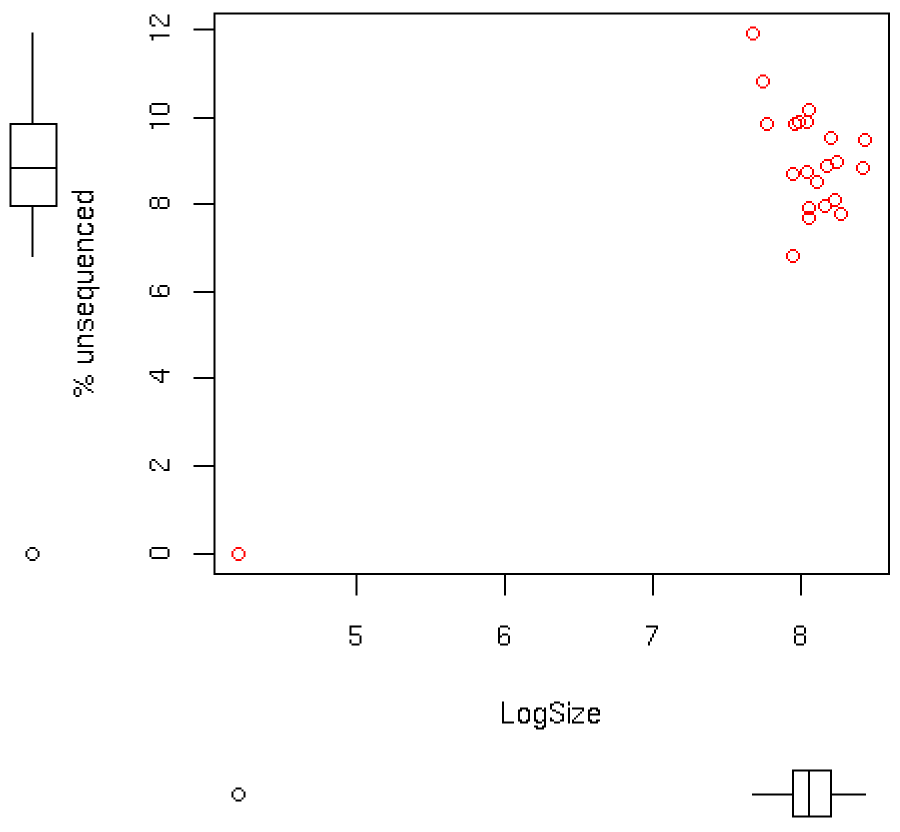

Rattus norvegicus

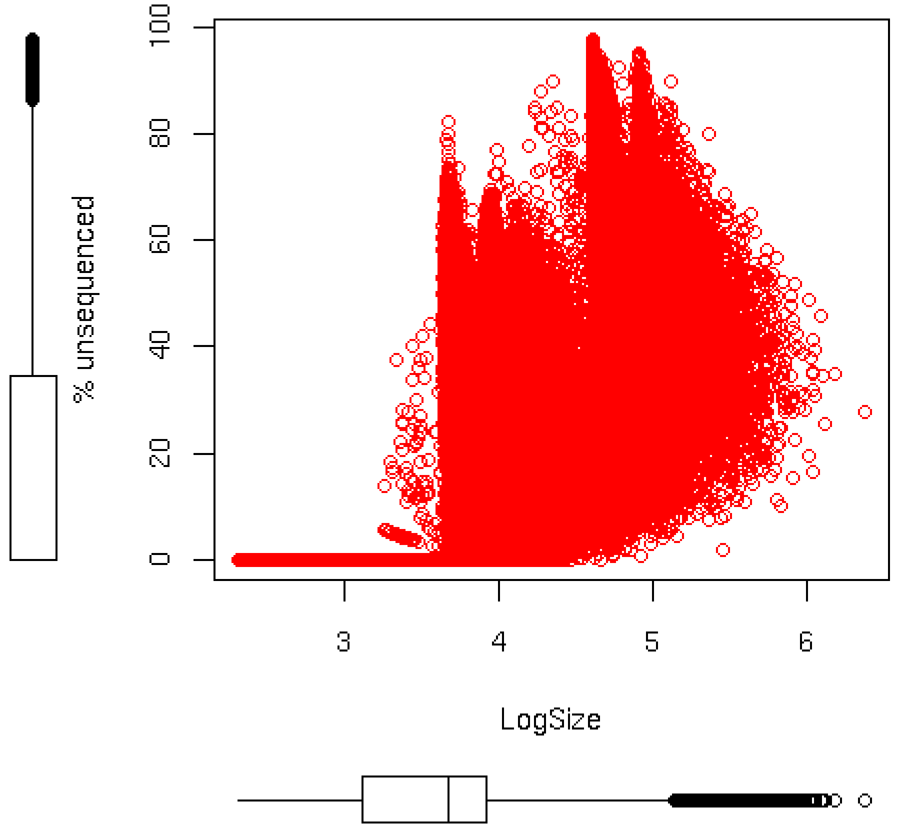

Sorex araneus

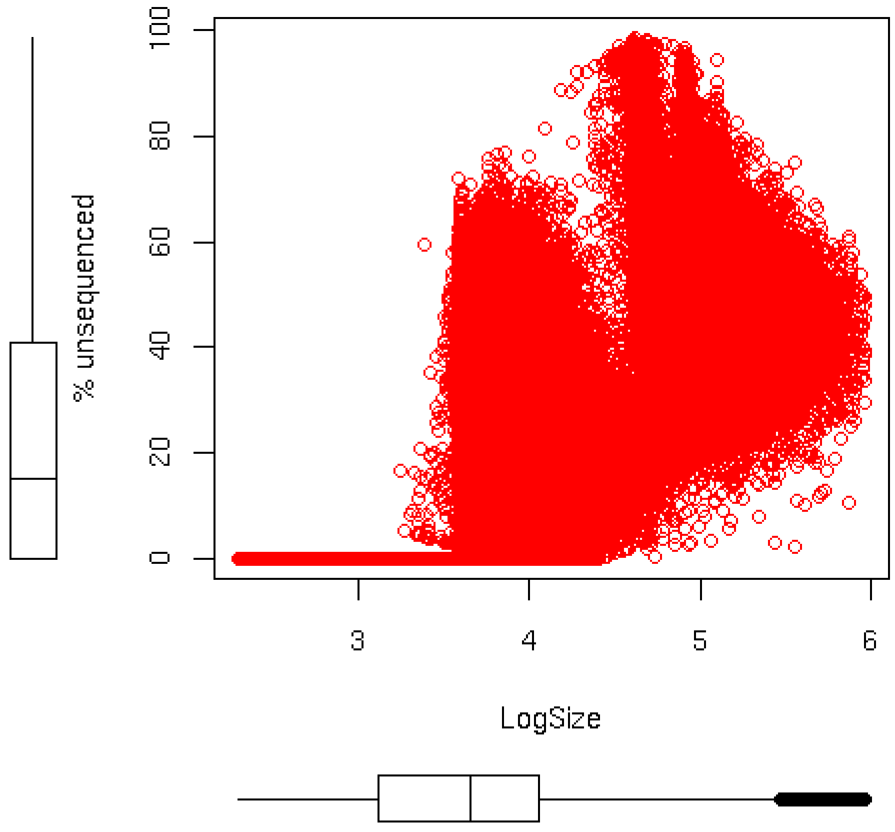

Spermophilus tridecemlineatus

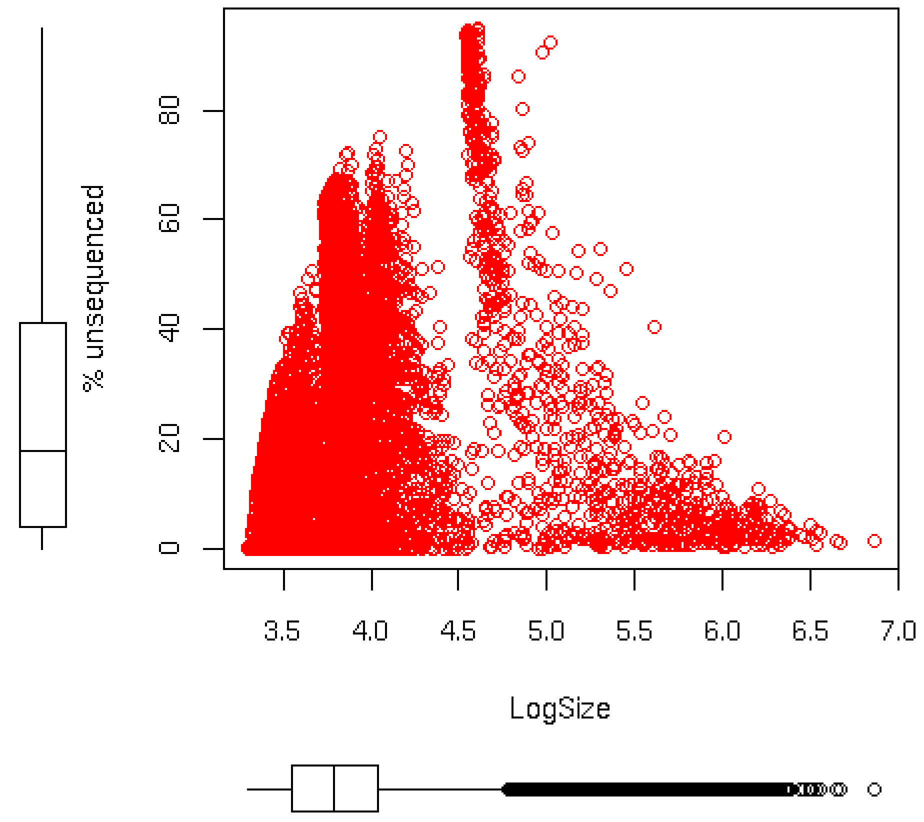

Takifugu rubripes

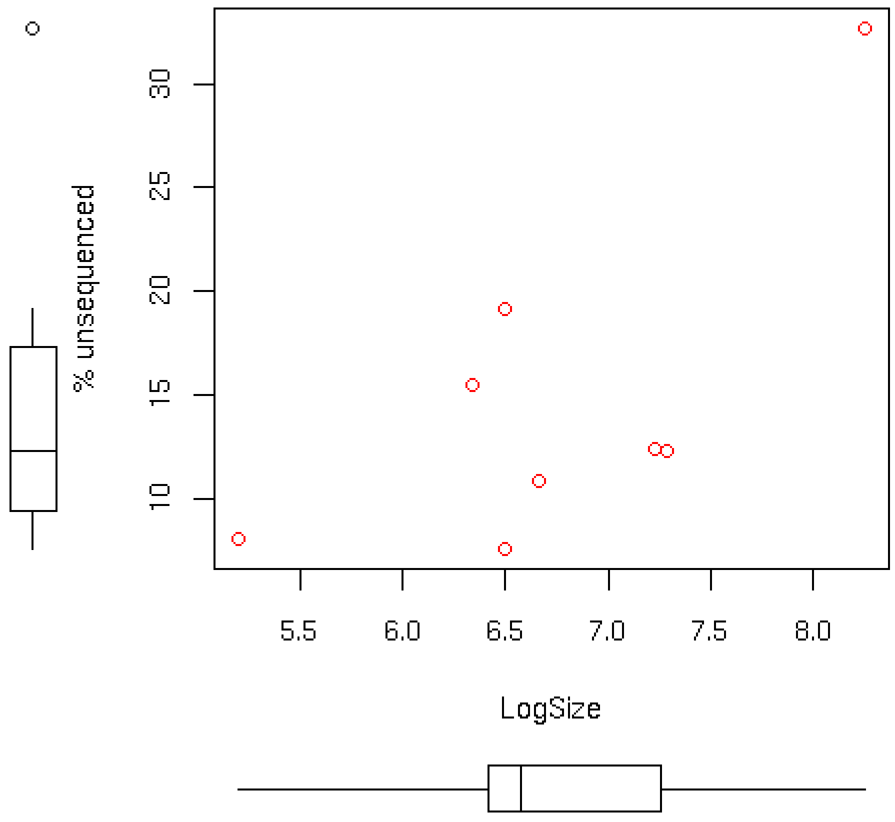

Tetraodon nigroviridis

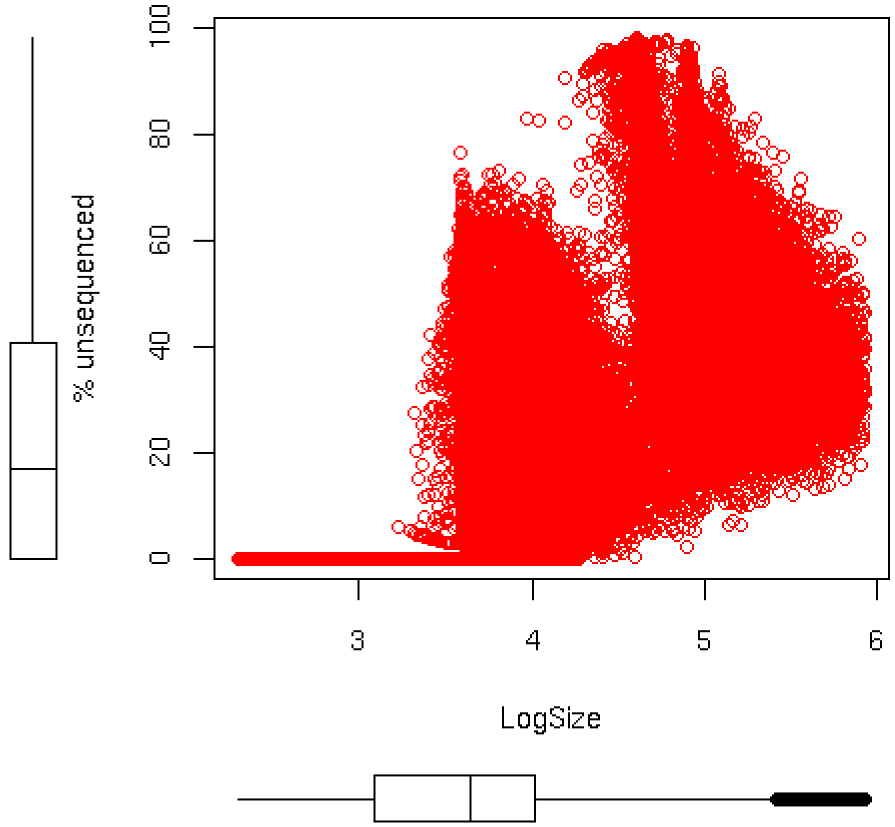

Tupaia belangeri

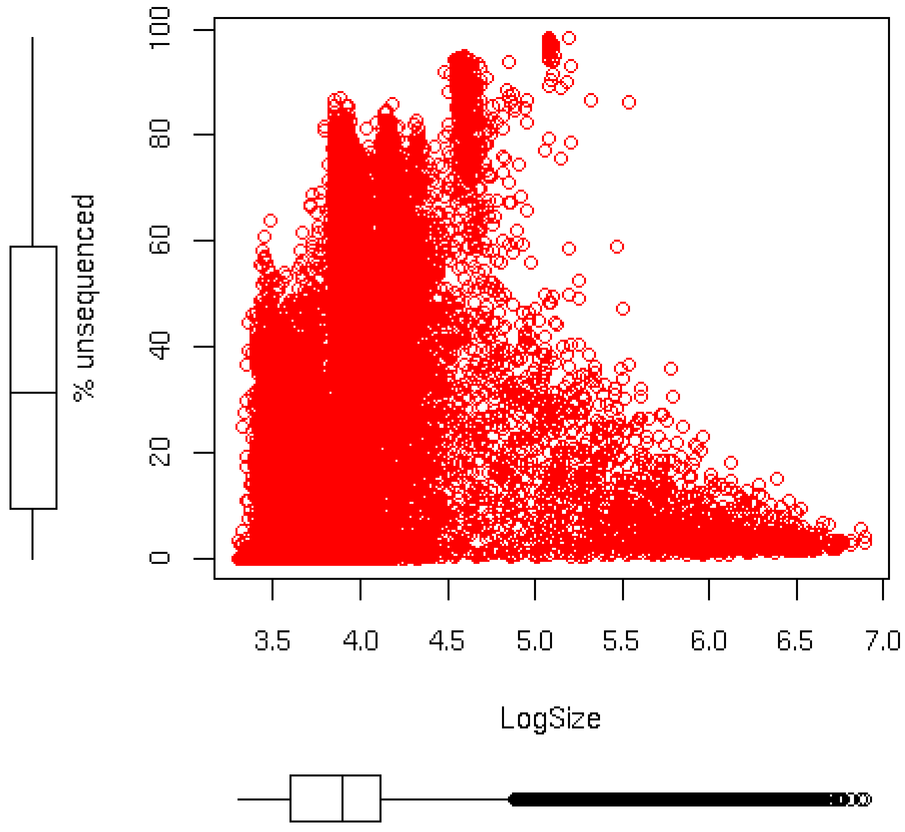

Xenopus tropicalis
